# Development and Validation of Case‐Finding Algorithms to Identify Periprosthetic Joint Infections After Total Hip Arthroplasty in Veterans Health Administration Data

**DOI:** 10.1002/pds.70311

**Published:** 2026-01-05

**Authors:** Jessica C. O'Neil, Yixuan Pei, Craig Newcomb, Randi Silibovsky, Judith A. O'Donnell, Charles L. Nelson, Evelyn Hsieh, Joseph King, Stephen Crystal, Jennifer S. Hanberg, Vincent Lo Re, Erica J. Weinstein

**Affiliations:** ^1^ Division of Infectious Diseases, Department of Medicine, Perelman School of Medicine University of Pennsylvania Philadelphia Pennsylvania USA; ^2^ Center for Real‐World Effectiveness and Safety of Therapeutics, Center for Clinical Epidemiology and Biostatistics, Department of Biostatistics, Epidemiology, and Informatics, Perelman School of Medicine University of Pennsylvania Philadelphia Pennsylvania USA; ^3^ Perelman School of Medicine at the University of Pennsylvania Philadelphia Pennsylvania USA; ^4^ Department of Orthopedic Surgery, Perelman School of Medicine The University of Pennsylvania Philadelphia Pennsylvania USA; ^5^ VA Connecticut Health System West Haven Connecticut USA; ^6^ Section of Rheumatology, Allergy and Immunology, Department of Medicine Yale University School of Medicine New Haven Connecticut USA; ^7^ Department of Neurosurgery, Yale University School of Medicine New Haven Connecticut USA; ^8^ Institute for Health, Health Care Policy, and Aging Research Rutgers University New Brunswick New Jersey USA; ^9^ Division of Rheumatology, Immunology and Inflammation Brigham and Women's Hospital Boston Massachusetts USA

**Keywords:** arthroplasty, case‐finding algorithm, electronic health records, joint replacement, periprosthetic joint infection, validation, Veterans Health Administration

## Abstract

**Purpose:**

To determine the positive predictive values (PPVs) of ICD‐9‐ and ICD‐10‐based diagnostic coding algorithms to identify periprosthetic joint infection (PJI) following total hip arthroplasty (THA) within the United States (US) Veterans Health Administration (VHA).

**Methods:**

We selected patients with: (1) any position hospital discharge ICD‐9 or ICD‐10 diagnosis of PJI, (2) ICD‐9, ICD‐10, or current procedural terminology (CPT) procedure codes for THA any time prior to PJI diagnosis, (3) CPT code for hip X‐ray within ±90 days of the PJI diagnosis, and (4) 1 or more CPT codes for arthrocentesis, arthrotomy, or revision arthroplasty all occurring within ±90 days of the PJI diagnosis date. We obtained separate samples of patients for ICD‐9 and ICD‐10‐based PJI diagnoses. These samples were stratified by THA medical center volume. Infectious disease physicians adjudicated each identified PJI event. The PPV (95% confidence interval [CI]) for the ICD‐9 and ICD‐10 PJI algorithms were calculated.

**Results:**

Among the 90 sampled hip PJI events for the ICD‐9 era, 79 were confirmed PJIs (PPV 87.8%, 95% CI 79.2%–93.7%). For the 90 sampled hip PJI events for the ICD‐10 era, 72 were confirmed PJIs (PPV 80.0%, 95% CI 70.3%–87.7%).

**Conclusion:**

These algorithms yielded a PPV of 87.8% (ICD‐9) and 80.0% (ICD‐10), for confirmed PJI events and could be considered for use in future pharmacoepidemiologic studies.

## Key Points

- Electronic healthcare databases may serve as valuable resources to study the pharmacoepidemiology of periprosthetic joint infection (PJI) following total hip arthroplasty (THA), but case finding algorithms to identify hip PJI have not been developed or validated within US healthcare databases.
- We developed and validated two case finding algorithms based on International Classification of Diseases, Ninth Revision (ICD‐9) and International Classification of Diseases, Tenth Revision (ICD‐10) diagnoses for hip PJI, in combination with Current Procedural Terminology (CPT) codes, to identify hip PJI within US Veterans Health Administration data.
- We identified possible hip PJI events based on the following criteria: (i) hospital discharge ICD‐9 or ICD‐10 diagnosis for PJI, (ii) ICD‐9, ICD‐10 or CPT procedure code for THA prior to PJI diagnosis, (iii) CPT code for hip X‐ray within ±90 days of the hip PJI diagnosis, and (iv) at least 1 CPT code for arthrocentesis, arthrotomy, or revision arthroplasty all occurring within ±90 days of the hip PJI diagnosis date.
- An algorithm consisting of an ICD‐9 or ICD‐10 code for PJI following a procedure code for THA, combined with CPT codes for a hip X‐ray and any one of arthrocentesis or arthrotomy within ±90 days of the PJI diagnosis date, had a positive predictive value (PPV) of 87.8% (ICD‐9) and 80.0% (ICD‐10), respectively, for confirmed hip PJI.
- These algorithms could be considered as fit for purpose for future pharmacoepidemiology studies of hip PJI within US Veterans Health Administration data.

## Plain Language Summary

Joint replacement surgery is very common and growing in the United States (US). PJI is a serious complication of joint replacement surgery. There are few large pharmacoepidemiological studies of hip PJI in the US. The Veterans Health Administration (VHA) is the largest integrated health system in the US and may be a valuable setting to study hip PJI. In order to study hip PJI within VHA data we developed four separate algorithms to identify patients who developed a hip PJI after undergoing hip replacement surgery. The algorithms used a combination of diagnosis codes and procedural codes to identify patients with a hip PJI. We then assessed the accuracy of each algorithm by having two independent physicians review 90 cases of hip PJI identified by the algorithm to confirm that a hip PJI truly occurred. We found that Algorithms 2A and 2B accurately identified hip PJI in greater than 80% of the cases.

## 1 Introduction

THA is a highly effective treatment for hip osteoarthritis [1, 2, 3] and one of the most common elective surgeries in the United States (US). Annual THA volume has risen steeply over the past two decades and is projected to reach 1.4 million in the US by 2040 [4]. Periprosthetic joint infection (PJI) is a dreaded complication of THA occurring in 1.0%–2.0% of patients [5]. As the volume of THA has grown, the total number of hip PJIs has risen as well [6, 7, 8]. PJI requires intensive and costly treatment [9]. Moreover, PJI negatively impacts patient mortality, well‐being, and quality of life [10, 11]. A recent US based population representative study of knee PJI has provided important insights into the incidence, determinants and microbiology of these infections in the US through the use of a validated case‐finding algorithm to accurately identify knee PJI [12, 13]. Our knowledge of the epidemiology and optimal pharmacologic therapies for prevention and treatment of hip PJI remains limited. The Veterans Health Administration (VHA) is the largest integrated healthcare system in the US and may be a valuable data source to examine important knowledge gaps in the use of antibiotics for the prevention and treatment of infection in these implanted medical devices [14]. However, in order to study the epidemiology of hip PJI, and investigate the real‐world impact of antimicrobial choice and duration on the prevention and treatment of these events, we must first develop and validate methods to accurately identify hip PJI within VHA data.

Few case finding algorithms for hip PJI have been developed and validated within electronic health record (EHR) databases, and even fewer in US‐based health systems. Hip PJI case finding algorithms have previously been developed and validated in a Danish prospective THA registry and a Canadian administrative database [15, 16]. Both algorithms identified hip PJI with reasonably high accuracy (PPV of 98% and 78%, respectively) by combining hip PJI diagnosis codes with additional data elements related to hip PJI care (procedural codes, microbiologic results). However, the performance of case‐finding algorithms can vary across populations and EHR databases [17]. The validity of algorithms to identify hip PJI within US EHR databases is unknown. One US‐based study demonstrated that International Classification of Diseases, Ninth Revision (ICD‐9) codes for hip PJI alone had a high degree of accuracy (PPV 87.5) in classifying PJI as an indication for revision THA among a population of patients undergoing revision THA in referral center [18]. However, this methodology: (i) fails to identify patients with PJI who do not undergo revision arthroplasty (up to 15% of patients with PJI do not undergo revision arthroplasty, and these patients are typically older and more medically complex) [19]; (ii) may have limited performance among community‐based settings where the majority of arthroplasty is performed in the US [20]; and (iii) has not been evaluated in the International Classification of Diseases, Tenth Revision (ICD‐10) era.

To address this methodologic gap, we developed and assessed the performance characteristics of case finding algorithms for hip PJI within VHA data, using ICD‐9 or ICD‐10 diagnoses in combination with relevant CPT codes.

## 2 Methods

### 2.1 Design and Data Source

We conducted our retrospective study between October 1, 1999 and September 30, 2022 using national VHA data. The VHA is the largest integrated healthcare system in the US, as of 2024, 9.1 million US veterans were enrolled in VHA care across 171 hospitals and 1400 clinics [14]. PJI diagnosis is complex, relying on a constellation of clinical, laboratory, microbiologic and pathologic findings [21]. VHA data are ideal for EHR‐based PJI research since they contain a broad array of elements from inpatient, outpatient, and emergency department encounters, including demographic information, medical diagnoses, surgical procedures, dispensed medications, provider notes, and results from laboratory, microbiologic, and pathologic tests.

### 2.2 Patient Selection for Validation

To evaluate the performance of approaches to identify hip PJI events within VHA data in both the ICD‐9 and ICD‐10 coding eras, we developed separate ICD‐9 and ICD‐10‐based algorithms for each approach, developing and evaluating a total of four algorithms. For each algorithm, we randomly sampled 90 patients with a potential hip PJI event for validation. Sampling was stratified by annual THA volume to allow for representation from low (< 20 THA annually), medium (20–49 THA annually), and high volume (> 49 THA annually) VHA hospitals.

Our first approach was adapted from previously validated algorithms within the VHA for knee PJI by Weinstein et al. [13], which identify knee PJI with a PPV of 75.0% (ICD‐9) and 85.0% (ICD‐10). These algorithms identify knee PJI based on: (i) hospital ICD‐9 or ICD‐10 discharge diagnosis (in principal or contributory position) of knee PJI; (ii) total knee arthroplasty (TKA) preceding the knee PJI diagnosis date; (iii) CPT code for a knee radiograph ±90 days from the knee PJI diagnosis date; and (iv) CPT code for arthrocentesis of a major joint, arthrotomy of a knee, blood culture, or other microbiologic procedure ±90 days from the PJI diagnosis date.

**FIGURE 1:**
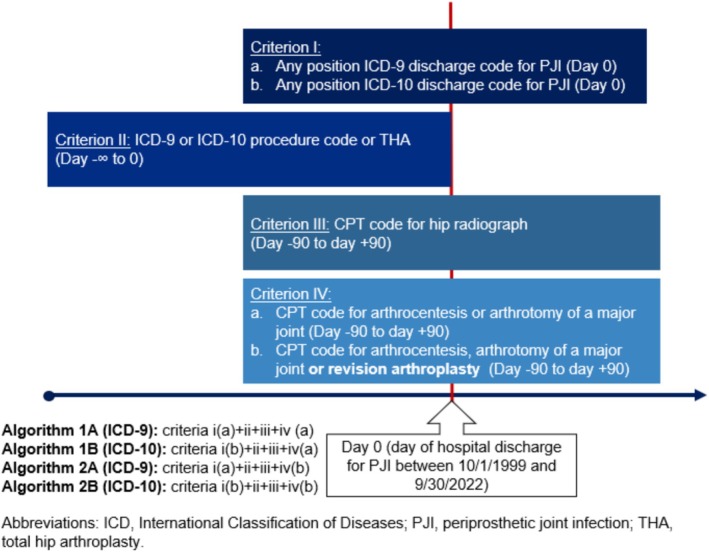
Design diagram highlighting selection of potential prosthetic joint infection events within the International Classification of Diseases, Ninth Revision (ICD‐9) era (Algorithms 1A and 2A) and International Classification of Diseases, Ninth Revision (ICD‐10) era (Algorithms 1B and 2B).

We modified these criteria for use in hip PJI (Figure 1) by identifying veterans who had: (i) hospital ICD‐9 (denoted Algorithm 1A) or ICD‐10 (denoted Algorithm 1B) discharge diagnosis (in principal or contributory position) of PJI (Table 1) recorded between October 1, 1999 and September 30, 2022; (ii) THA ICD‐9 (81.51) or ICD‐10 (0SRBxxx, 0SR9xxx) procedure code prior to the PJI diagnosis; (iii) CPT code for a hip radiograph ±90 days from the hip PJI diagnosis date; and (iv) CPT code for arthrocentesis of a major joint, arthrotomy of a hip ±90 days from the PJI diagnosis date. For criterion iv, we eliminated the blood culture or other microbiologic culture elements based on preliminary observation that a significant number of superficial surgical site infections were being identified as possible hip PJI events by including these elements. We were confident in the use of diagnosis codes for identification of a preceding primary THA (criterion ii) as ICD‐9 codes have previously been shown to identify this procedure accurately within VHA data, with a PPV of 98% [22].

**TABLE 1:** Periprosthetic joint infection‐related diagnosis codes and descriptions.

Code type	Code number	Code description
ICD‐9 and ICD‐10 diagnoses for periprosthetic joint infection		
ICD‐9	996.60	Infection and inflammatory reaction due to internal periprosthetic device implant and graft
	996.66	Infection and inflammatory reaction due to unspecified internal joint prosthesis
	996.67	Infection and inflammatory reaction due to other internal orthopedic device, implant, and graft
	996.69	Infection and inflammatory reaction due to other internal periprosthetic device, implant, and graft
ICD‐10	T84.50XA	Joint or orthopedic prosthesis infection unspecified prosthesis, initial encounter
	T84.49XA	Joint or orthopedic prosthesis infection unspecified prosthesis, initial encounter
	T84.7XXA	Joint or orthopedic prosthesis infection unspecified prosthesis, initial encounter
	T84.51XA	Infection and inflammatory reaction due to internal right hip prosthesis, initial encounter
	T84.52XA	Infection and inflammatory reaction due to internal left hip prosthesis, initial encounter
	T81.42XA	Infection following a procedure, deep incisional surgical site, initial encounter
	T81.43XA	Infection following a procedure, organ and space surgical site, initial encounter
	T85.79XA	Infection due to other internal periprosthetic devices, implants and grafts, initial encounter
Current procedural terminology codes for hip X‐ray		
CPT	73 500	X‐ray hip 1 view
	73 510	X‐ray hip 2 views
	73 501	X‐ray hip unilateral with pelvis 1 view
	73 502	X‐ray hip L/R with or without pelvis minimum 2–3 views
	73 503	X‐ray hip L/R with or without pelvis minimum 4 views
	73 520	X‐ray hips bilateral
	73 521	X‐rat hips bilateral with pelvis 2 views
	73 522	X‐ray hips bilateral with pelvis 3–4 views
	75 323	X‐rat hips bilateral with pelvis minimum 5 views
Current procedural terminology codes for relevant surgical procedures		
CPT	20 610	Arthrocentesis, aspiration and/or injection; major joint
	20 611	Arthrocentesis, aspiration and/or injection, major joint, ultrasound guided
	27 030	Arthrotomy, hip with drainage
	27 033	Arthrotomy, hip, including exploration, or removal of loose foreign body
	27 052	Arthrotomy with biopsy, hip joint
	27 054	Arthrotomy, with synovectomy, hip joint
	27 134	Revision of total hip arthroplasty; both components, with or without autograft or allograft
	27 137	Revision of total hip arthroplasty; acetabular component only, with or without autograft or allograft
	27 138	Revision of total hip arthroplasty; femoral component only, with or without allograft
	27 190	Removal of hip prosthesis
	27 091	Removal of prosthesis, including total hip prosthesis, methylmethacrylate with or without insertion of spacer, knee

Abbreviations: CPT, current procedural terminology; ICD‐9, International Classification of Diseases, 9th revision; ICD‐10, International Classification of Diseases, 10th Revision.

In an attempt to increase the performance of our algorithm, we conducted a second approach whereby we amended criterion iv to also include CPT codes for revision arthroplasty of a hip ±90 days from the PJI diagnosis date (Table 1) in addition to those who had an arthrotomy or arthrocentesis during that time period. The revision of criterion iv was denoted as Algorithm 2A for the ICD‐9 era and Algorithm 2B for the ICD‐10 era. This revision was intended to include those patients who did not undergo a diagnostic arthrocentesis or arthrotomy prior to a revision arthroplasty, but rather just underwent revision arthroplasty alone. For the diagnosis of knee PJI, it is common to undergo an outpatient knee arthrocentesis procedure preceding a revision arthroplasty to sample synovial fluid and evaluate for inflammation and infection [8]. However, hip arthrocentesis is more technically challenging and less commonly performed. Therefore, a significant portion of these patients proceed to revision arthroplasty without preceding sampling of synovial fluid and would therefore not be identified by our first approach.

### 2.3 PJI Definition

We applied the clinical case definitions (Table 2) developed by Weinstein et al. [12] to classify possible PJI events identified as: no PJI, probable PJI, or definite PJI. This case definition schema was chosen because the criteria for definite PJI closely aligns with the widely adopted 2013 International Consensus Group Definition for PJI and the 2011 Musculoskeletal Infection Society Definition of PJI, the first established consensus definition of PJI [21, 23]. Major and minor criteria to classify PJI are specified in Table 2. Definite PJI required the presence of one of two major criteria or three of five minor criteria. Probable PJI required two of five minor criteria plus a dispensed fill for an antibiotic on the date of the PJI event for at least 4 weeks' duration. Patients were classified as having a PJI event if they met a definite or probable definition for PJI. We included probable PJI in our case definition because elements necessary to classify a possible PJI event as definite may be absent in our retrospective study due to failure to obtain laboratory, microbiology or pathology samples or initiation of empiric antibiotic prior to microbiologic sampling leading to culture sterilization. Events that did not meet criteria for definite or probable PJI were classified as no PJI.

**TABLE 2:** Case definition of prosthetic joint infection.

Definite PJI	Probable PJI
Any *one* of the following criteria present during admission or within 30 days prior to admission: Two positive periperiprosthetic cultures with phenotypically identical organisms A sinus tract communicating with the joint *Three or more* the following given findings present during admission or within 30 days prior to admission: Serum C‐reactive protein (CRP) of ≥ 10 mg/L AND erythrocyte sedimentation rate (ESR) ≥ 30 mm/h Synovial fluid white blood cell (WBC) count of ≥ 3000 cells/μL OR + or ++ change on leukocyte esterase test strip Synovial fluid polymorphonuclear neutrophil percentage (PMN%) of ≥ 80% > 5 neutrophils per high power field in five high power fields (×400) or acute inflammation reported on pathology A single positive synovial fluid culture	*Two* or more of the following: Serum C‐reactive protein (CRP) of ≥ 10 mg/L AND erythrocyte sedimentation rate (ESR) ≥ 30 mm/h Synovial fluid white blood cell (WBC) count of ≥ 3000 cells/μL OR + or ++ change on leukocyte esterase test strip Synovial fluid polymorphonuclear neutrophil percentage (PMN%) of ≥ 80% > 5 neutrophils per high power field in five high power fields (×400) or acute inflammation reported on pathology A single positive synovial fluid culture AND Prescription for antibiotic at time of PJI event, for minimum of 4‐week course

### 2.4 Confirmation of Outcomes

An infectious diseases physician (E.J.W.) trained an abstractor to identify and input relevant data elements for defining definite and probable PJI (Table 2) into a VHA‐hosted Research Electronic Data Capture (REDCap) form (Data S1) [24]. Abstractor training consisted of a review of the diagnosis and management of PJI, an overview of the VHA EHR interface, an explanation of the relevant language in clinician and operative notes (i.e., sinus tract, arthrocentesis), and practice in chart abstraction. The trained abstractor then identified data elements from clinician notes (admission notes, progress notes, discharge summaries), operative reports and laboratory, microbiology, and pathology results. In order to assure accurate abstraction of the data elements, E.J.W. and the trained abstractor independently completed abstraction of the first 10 patients and compared results with high concordance.

Two infectious diseases clinicians (E.J.W. and J.C.O.) independently reviewed each abstraction form to classify each possible hip PJI event as definite, probable, or no PJI. Each possible hip PJI event was adjudicated based on the components of the abstraction form alone and adjudicators were blinded to the patient medical record or further information such as the clinical impressions of the providers that had cared for the patient during the possible hip PJI event. Each element of the case definition had to be present during or within 30 days prior to the hip PJI hospitalization. We intended to review any disagreements with a third infectious diseases clinician to arbitrate the event; however, after review of the PJI case definition, no disagreement occurred between the reviewers.

### 2.5 Statistical Analyses

For each algorithm, we calculated the PPV with 95% confidence interval (CI) for probable or definite PJI using the Clopper–Pearson interval [25]. We selected PPV as our primary outcome of interest because a sufficiently high PPV implies that identified outcomes represent true events. We estimated that a sample of 90 PJI events for each algorithm would allow determination of the PPV with a sufficiently narrow 95% CI width of ±10%, assuming a PPV of 80%. We also determined inter‐rater reliability by calculating the percent agreement and kappa statistic between the two adjudicators. Data analysis was conducted with Stata 18.1 (Stata Corp, College Station, TX).

## 3 Results

We identified 60 098 primary THAs in the VHA between October 1, 1999 and September 31, 2022. Of these 450 met criteria for Algorithms 1A or 1B, and 974 met criteria for Algorithms 2A or 2B. For each of the four algorithms, 90 patients meeting the relevant algorithm criteria were sampled. The demographic characteristics of the patients who met criteria for the algorithm and who were sampled are described in Data S2. Sampled hip PJI events were evenly distributed across years of the study period for Algorithm A1, A2, B1, and B2. By stratifying our sampling by VHA hospital THA volume, we achieved representation of hip PJI from low, medium and high THA volume hospitals. Medical records were available for all sampled possible PJI events, and every sampled event was independently adjudicated by E.J.W. and J.C.O.

Among the 90 sampled hip PJI events for Algorithm 1A (Table 3), 71 were confirmed to have a hip PJI (68 definite, 3 probable) yielding a PPV of 78.9% (95% CI 70.5%–87.3%). For the 90 sampled hip PJI events for Algorithm 1B, 68 were confirmed to have a hip PJI (63 definite, 5 probable) yielding a PPV of 75.6% (95% CI 66.7%–84.5%). The percent agreement among adjudicators for both algorithms was 100%, yielding a kappa statistic of 1.0.

**TABLE 3:** Positive predictive values (with 95% confidence intervals) of case‐identifying algorithms to identify hip periprosthetic joint infection.

Algorithm (ICD era)	Number of confirmed PJI	PPV (95% CI)	Number that did not meet criteria	Reason not confirmed						
				Hip PJI not meeting case definition for probable or definite PJI	Superficial surgical site infection	Other hardware infection	Second stage of two stage revision	Native joint infection	Other infection	Noninfectious complication
1A (ICD 9)	71	78.9% (70.5%–87.3%)	19	10	0	3	0	2	2	2
1B (ICD10)	68	75.6% (66.7%–84.5%)	22	12	3	0	3	2	0	2
2A (ICD 9)	79	87.8%, CI (79.2%–93.7%)	11	9	0	0	0	0	1	1
2B (ICD10)	72	80.0% (70.3%–87.7%)	18	15	1	0	1	1	0	0

In contrast, Algorithms 2A and 2B (addition of revision arthroplasty to criterion iv) demonstrated the higher PPVs. Among the 90 samples hip PJI events for Algorithm 2A (Table 3), 79 were confirmed to have a hip PJI (65 definite, 14 probable) yielding a PPV 87.8% (95% CI 79.2%–93.7%). For the 90 sampled hip PJI events for Algorithm 2B, 72 were confirmed to have a hip PJI (64 definite, 8 probable) yielding a PPV of 80.0% (95% CI 70.3%–87.7%). The percent agreement among adjudicators for both algorithms was again 100% yielding a kappa statistic of 1.0.

Finally, when comparing confirmed hip PJIs between Algorithm 1A/1B and 2A/2B, all confirmed cases identified by Algorithms 1A and 1B would have been identified by Algorithms 2A and 2B, respectively. Of the 79 confirmed hip PJIs identified by Algorithm 2A, only 36 (45.6%) also met the criteria for Algorithm 1A. Of the 72 confirmed hip PJIs identified by Algorithm 2B, only 23 (32.0%) also met the criteria for Algorithm 1B.

Table 3 indicates the most common reasons that sampled hip PJI events were not categorized as definite or probable for each algorithm. For all algorithms, the most common reason that possible PJI events were categorized as nonhip PJI events was due to insufficient laboratory, microbiology, or pathology data. Other reasons for included events attributed to admissions for re implantation of a permanent arthroplasty following treatment for a preceding hip PJI (i.e., second stage of a two‐stage revision), other orthopedic hardware infection (knee PJI, infected intramedullary nail), native hip septic arthritis, other infections (urinary tract infection, bacteremia) and noninfectious complications (post operative hematoma, aseptic loosening).

## 4 Discussion

In this study, we developed and validated two ICD‐9 and two ICD‐10 based algorithms using a combination of diagnosis and procedural codes to identify PJI diagnoses in VHA data. Algorithms 1A (ICD‐9) and 1B (ICD‐10) incorporated: (i) PJI diagnosis codes for PJI, (ii) a preceding THA procedure code, (iii) a procedure code for hip radiograph within 90 days of PJI diagnosis date, and (iv) procedure for arthrocentesis or arthrotomy of hip or major joint within 90 day of diagnosis date. These algorithms performed reasonably well, achieving PPVs of 78.9% (ICD‐9) and 75.6% (ICD‐10), for Algorithms 1A and 1B, respectively. However, these Algorithms identified only 450 hip PJI events out of 60 098 primary hip arthroplasties. A hip PJI rate of 0.7% is below the 1%–2% reported in existing literature [5]. To enhance identification of hip PJI events, we revised criteria iv within Algorithms 2A (ICD‐9) and 2B (ICD‐10] to include procedure codes for revision arthroplasty in addition to arthrocentesis and arthrotomy. This modification yielded both an increase in the rate of identification of hip PJI events (974 hip PJI events, hip PJI rate of 1.6%) and an improved PPV for identification of hip PJI events in both the ICD‐9 (Algorithm 2A: PPV 87.8%, 95% CI 79.2%–93.7%) and ICD‐10 eras (Algorithm 2B: PPV 80.0%, 95% CI 70.3%–87.7%).

Our algorithms were adapted from the knee PJI case finding algorithm developed and validated by Weinstein et al. [13] within the same data source. The performance of our hip PJI algorithms mirrors the performance of their knee PJI algorithms (PPVs of 75.0% in ICD‐9 and 85.0% in ICD‐10) [13], which have already been implemented to study the incidence, determinants, and microbiology of knee PJI [12]. Our algorithm differs from that of Weinstein et al. [13] with the addition of revision arthroplasty as described above as well as the exclusion of blood cultures or microbiologic cultures not otherwise specific from criterion iv based on preliminary observations that a significant number of superficial surgical site infections were being inappropriately identified as possible hip PJI. This is perhaps due reduced accuracy of ICD coding within the VHA to distinguish PJI from a superficial surgical site infection in the hip as compared to the knee. Algorithms 2A and 2B performed superiorly to a case finding algorithm developed using an administrative database from four Canadian tertiary care hospitals (PPV 78%, 95% CI 74.0%–82.0%) [15]. The Canadian algorithm relied on procedural codes placement of a central venous catheter (CVC) to identify patients with PJI for use of long‐term intravenous antibiotics. Algorithms requiring procedure codes for revision arthroplasty or CVC placement will exclude those patients with PJI who are treated oral antibiotics, an increasingly common therapeutic approach [19, 26, 27]. Our Algorithms 2A and 2B did not achieve the performance of a case finding algorithm developed in a prospective Danish hip arthroplasty registry (PPV 98%) [15]. While there are efforts in the US to establish a prospective arthroplasty registry, the US registry does not currently capture granular microbiologic and antimicrobial data available in the VHA administrative database which are crucial to future pharmacoepidemiologic research in this space [28]. Furthermore, both the US and Danish registries require revision arthroplasty for identification of PJI cases. These approaches do not identify patients with PJI who are not candidates for revision arthroplasty (often older or more medically complex patients) or who are treated with antimicrobials alone [29]. In contrast our Algorithms 2A and 2B identify patients with PJI who do not undergo revision arthroplasty. While this may only represent a small subset of PJI cases, patients who are not candidates for revision surgery face higher rates of treatment failure [29]. Further research is therefore crucial to determine optimal antimicrobial selection and duration for this challenging clinical scenario.

Our study has several strengths. We applied a rigorous case definition built from international consensus [13] to classify probable and definite PJI. In fact, the majority of possible PJI events that were classified by our adjudicators as no PJI were still thought to be attributable to a hip PJI, but not meeting our international consensus‐based definition. We developed and validated algorithms in both the ICD‐9 and ICD‐10 era to allow for use in longitudinal epidemiologic studies of hip PJI across multiple recent decades. Because the criteria for our case definitions had to be present within 30 days prior to or during the PJI hospital admission, our algorithm also establishes the date of PJI diagnosis with a reasonable confidence. An additional strength of our study is that our algorithms may be employed in combination with the Weinstein et al. algorithm in large US‐based population representative studies to compare and contrast incidence, determinants and outcomes of hip and knee PJI as well as to determine optimal antimicrobial therapies for the prevention and treatment of PJI.

Our study has several potential limitations. First, misclassification of PJI outcomes is possible. This risk was mitigated by employing two independent infectious diseases physician to adjudicate PJI events using a standardized case definition with objective criteria. It is possible that missing data may have contributed to misclassification of PJI events, since absent (either uncollected or unreported) laboratory, microbiologic, or pathologic results were considered not meeting criteria for our case definition and may have led to undercounting of probable or definite PJI. Second, while our algorithms can reasonably establish a date of PJI diagnosis, hip PJI can be an indolent, slowly progressive infection and our algorithms cannot determine date of PJI onset. Third, we did not measure the negative predictive value (NPV), sensitivity, or specificity of these algorithms. We would expect the NPV to be high given the low incidence of PJI [30]. It was not feasible to determine the sensitivity or specificity given the absence of a longitudinal registry of THA outcomes and confirmed PJI diagnoses within VHA data. While we did not determine the sensitivity of these algorithms, we did demonstrate that by including revision arthroplasty to criteria iv of Algorithm 2A/2B, we considerably increased the number of hip PJI events identified while also increasing the PPV as compared to Algorithms 1A/1B. Fourth, the performance of our algorithms may vary in other US‐based EHR databases and should be evaluated before applying this methodology to a different data source.

In conclusion, we developed and validated separate ICD‐9 and ICD‐10 based case finding algorithms to identify hip PJIs within VHA data. Our algorithms, comprised of inpatient PJI diagnosis codes, a preceding THA code, and procedure codes for a hip radiograph, and an arthrocentesis, arthrotomy or revision arthroplasty identified PJI diagnoses with ≥ 80% PPV. These algorithms could be used alone or in combination with a previously validated knee PJI algorithm [13] in future pharmoacoepidemiologic studies to advance our understanding of this devastating complication of arthroplasty and determine optimal antimicrobial therapy for PJI prevention and treatment.

## Funding

This work is supported by the National Institute of General Medical Sciences Clinical Pharmacoepidemiology Training Program (T32‐GM075766), the National Institute on Alcohol Abuse and Alcoholism and (P01 AA029545, U24 AA020794). This material is the result of work supported with resources and the use of facilities at the Corporal Michael J. Crescenz Philadelphia VA Medical Center. The views and opinions expressed in this manuscript are those of the authors and do not necessarily represent those of the Department of Veterans Affairs or the United States government.

## Ethics Statement

This study was approved by the institutional review boards of Yale University, VA Connecticut Healthcare System and the Corporal Michael J. Crescenz Department of VA Medical Center. It has been granted a waiver of informed consent and is compliant with the Health Insurance Portability and Accountability Act.

## Conflicts of Interest

The authors declare no conflicts of interest.

## Supporting information


**Data S1:** pds70311‐sup‐0001‐AppendixA.pdf.

**Data S2:** pds70311‐sup‐0002‐AppendixB.docx.
