# Supplementary material for: Development and Validation of Case‐Finding Algorithms to Identify Periprosthetic Joint Infections After Total Hip Arthroplasty in Veterans Health Administration Data
Source: Pharmacoepidemiol Drug Saf. 2026 Jan 5;35(1):e70311. doi: 10.1002/pds.70311 (PMC12768563; doi:10.1002/pds.70311)
Supplement: Supplementary file 1 — Data S1: pds70311‐sup‐0001‐AppendixA.pdf. [file PDS-35-e70311-s001.pdf]

Abstraction Form

Record ID ____________________

Which algorithm?
(If PJI was diagnosed before Oct 1, 2015, then ICD-9, if after, then ICD-10)

◯ ICD 9
◯ ICD 10

General Instructions

Provide a response for each question. The instructions for each section will direct you to the appropriate materials to complete that section. A "Yes" response is for a definite diagnosis (e.g., not preceded by "probable," "possible," or "rule out"). If you are uncertain about a response, or if a condition is written in a physician's note but the diagnosis is not confirmed, indicate "Unconfirmed." Use the optional memo box at the end of each section for any comments, questions, or concerns.

Section A. Confirmation of prior THA and Clinician Diagnosis of PJI During Hospitalization

History of THA prior to PJI event?

◯ No
◯ Yes
◯ Unconfirmed

Does this infection episode correspond to an infection in a hip that has been replaced?

◯ No
◯ Yes
◯ I don't know

Which of the following best describes the infection event:

◯ Infection was in a native hip joint
◯ Infection was in a prosthetic knee joint
◯ Infection was in a prosthetic shoulder joint
◯ Infection was in a prosthetic ankle joint
◯ Infection was associated with orthopedic hardware but not true joint replacement (i.e. intramedullary nail or screws)
◯ Infection was in an alternative non-orthopedic medical implant such as abdominal mesh, cardiac device, etc
◯ Other

Documentation in clinical notes of sinus tract communicating with prosthetic hip joint?

◯ No
◯ Yes
◯ Unconfirmed

Did patient have surgical procedure (debridement, poly exchange, single stage revision, multiple stage revision)? This excludes diagnostic arthrocentesis

◯ No
◯ Yes
◯ Unconfirmed

Was the patient discharged with an antibiotic prescription for ≥4 weeks for the indication of PJI?

◯ No
◯ Yes
◯ Unconfirmed

If the patient was deemed by physicians not to have a PJI, what alternate diagnosis were they given? Examples can include cellulitis or superficial infection, gout, traumatic fracture, etc. ______________________________

Comments

Use this space to provide any comments, questions, or concerns regarding the items in this section. (Optional) ______________________________

Section B. Laboratory Criteria Indicative of a PJI event.

If infection is deemed not to be in the hip replacement, then can skip answering this section as it is not relevant.

Using the physician's notes and laboratory results, indicate if the patient was reported to have any of the following laboratory tests performed and their results during hospitalization or documented within 30 days prior to admission for PJI. All the tissue and arthrocentesis-specific answers correspond only to HIP samples

CRP ≥ 10mg/L or 1.0mg/d?
- ◯ No
- ◯ Yes
- ◯ Not performed

Highest recorded CRP ______________________________ (mg/d or mg/L (specify))

ESR ≥ 30mm/hr?
- ◯ No
- ◯ Yes
- ◯ Not performed

Highest recorded ESR ______________________________ (mm/hr)

Was a hip arthrocentesis or synovial fluid sampling performed (ok if was sent at time of surgery)?
- ◯ No
- ◯ Yes

Synovial fluid white blood cell (WBC) count of ≥ 3,000 cells/microL?
- ◯ No
- ◯ Yes
- ◯ not performed

Highest recorded synovial WBC value ______________________________ (cells/microL)

Synovial fluid polymorphonuclear neutrophil percentage (PMN%) of ≥80% ?
- ◯ No
- ◯ Yes
- ◯ not performed

Highest PMN % in synovial fluid ______________________________

Synovial fluid leukocyte esterase + or ++?
◯ No
◯ Yes
◯ Not performed

>5 neutrophils per high power field in 5 high power fields (x400) or acute inflammation reported on surgical pathology?
◯ No
◯ Yes
◯ Not performed

Comments

Use this space to provide any comments, questions, or concerns regarding the items in this section. (Optional)
____

Section D. Microbiologic Results Indicative of Infection

Using the physician's notes and microbiology data, indicate if the patient was reported to have any of the following microbiologic tests collected during the hospitalization or 30 days prior to admission.

Was an arthrocentesis culture sent? (may be labeled body fluid culture, synovial fluid, hip fluid, etc)
◯ No
◯ Yes
◯ Not confirmed

Arthrocentesis culture result:
◯ Negative
◯ Positive
◯ Unconfirmed

Arthrocentesis culture organism(s):
____

How many cultures were sent intra-operatively?
◯ 0
◯ 1
◯ 2
◯ 3
◯ 4
◯ 5+
(Select 0 if no surgery done)

Intraoperative culture results
◯ Negative
◯ Positive
◯ Unconfirmed

Intraoperative culture organism(s)
____

Total # of periprosthetic cultures with phenotypically identical organism(s): (Includes synovial fluid and tissue cultures/surgical cultures)
◯ 0
◯ 1
◯ 2
◯ 3
◯ 4
◯ 5+
(If polymicrobial, select option with highest number (both arthrocentesis and operative cultures))

Comments

Use this space to provide any additional comments (Optional)
