# Supplementary material for: Development and Validation of Case‐Finding Algorithms to Identify Periprosthetic Joint Infections After Total Hip Arthroplasty in Veterans Health Administration Data
Source: Pharmacoepidemiol Drug Saf. 2026 Jan 5;35(1):e70311. doi: 10.1002/pds.70311 (PMC12768563; doi:10.1002/pds.70311)
Supplement: Supplementary file 2 — Data S2: pds70311‐sup‐0002‐AppendixB.docx. [file PDS-35-e70311-s002.docx]

Appendix B: Baseline characteristics of all possible PJI events and sampled PJI events by algorithm

|  | **All Identified by Algorithms 1A & 1B (n = 450)** | **All Identified by Algorithms 2A & 2B (n=974)** | **All Sampled Subsets for Algorithms 2A (n=90)** | **All Sampled Subsets for Algorithms 2B**  **(n=90)** |
| --- | --- | --- | --- | --- |
| **Age, median, (IQR)** | 67.6 | 64.1 | 64 (58-73) | 68 (59-73) |
| **Male Sex, n (%)** | 423 (94.1%) | 927 (95.2%) | 84 (93.3%) | 85 (94.4%) |
| **Race/ Ethnicity, n (%)**  **Non-Hispanic Black**  **Non-Hispanic White**  **Hispanic**  **Other**  **Unknown** | 68 (15.1%)  321 (71.3%)  22 (4.9%)  28 (6.2%)  11 (2.4%) | 137 (14.1%)  690 (70.8%)  51 (5.2%)  75 (7.7%)  21 (2.2%) | 10 (11.1%)  68 (75.6%)  4 (4.4%)  6 (6.7%)  2 (2.2%) | 17 (18.9%)  64 (71.1%)  1 (1.1%)  4 (4.4%)  4 (4.4%) |
| **Year of PJI Event, median, (IQR)** | 2012 (2009-2014) | 2019 (2017-2021) | 2012 (2009-2014) | 2019 (2017-2021) |

Abbreviations: IQR, interquartile range; PJI, periprosthetic joint infection
